# Ten-year follow-up after combined coronary artery bypass grafting and transmyocardial laser revascularization in patients with disseminated coronary atherosclerosis

**DOI:** 10.1007/s10103-018-2514-9

**Published:** 2018-05-07

**Authors:** Janusz Konstanty-Kalandyk, Jacek Piątek, Anna Kędziora, Krzysztof Bartuś, Rafał Drwila, Tomasz Darocha, Grzegorz Filip, Bogusław Kapelak, Bryan HyoChan Song, Jerzy Sadowski

**Affiliations:** 1 Department of Cardiovascular Surgery and Transplantology, John Paul II Hospital, Jagiellonian University, ul. Pradnika 80, 31-202 Cracow, Poland; 2 Department of Anesthesiology and Intensive Care, John Paul II Hospital, Jagiellonian University, ul. Pradnika 80, 31-202 Cracow, Poland; 3 Jagiellonian University, sw. Anny 12, Cracow, Poland

**Keywords:** Coronary artery disease, Disseminated coronary atherosclerosis, Transmyocardial laser revascularization, Coronary artery bypass grating

## Abstract

Coronary artery disease involving heavily calcified lesions has been associated with worse short- and long-term outcomes including increased mortality. This paper aims to evaluate long-term survival benefit when CABG + transmyocardial laser revascularization (TMLR) are performed on the hearts of patients with disseminated coronary atherosclerosis (DCA). This novel retrospective study was conducted between 1997 and 2002 and followed 86 patients with ischemic heart disease and severe DCA who underwent TMLR using a Holmium:YAG laser and/or CABG. There were 46 patients who had CABG plus TMLR on at least one heart wall (“combined therapy group”) and 40 patients who had CABG or TMLR separately on at least one heart wall (“single therapy group”). For the whole group, actuarial survival at 10 years was 78.3% in the combined group compared to 72.5% in the single therapy group (*p* = 0.535). Ten-year survival in the combined vs. single therapy group for the anterior heart walls was 100 vs. 72.2% (*p* = 0.027). For the lateral and posterior heart walls were 73.7 vs. 73.3% (*p* = 0.97) and 84.2 vs. 72% (*p* = 0.27), respectively. Kaplan-Meier survival analysis showed benefit only for the anterior heart wall (F Cox test, *p* = 0.103). Single therapy procedures on all heart walls (odds ratio 1.736, *p* = 0.264) or on the anterior heart wall only (odds ratio 3.286, *p* = 0.279) were found to be predictors of 10-year late mortality. Combined therapy (TMLR + CABG) provides benefit for perioperative mortality and long-term survival only when provided on the anterior heart wall. For patients with disseminated coronary atherosclerosis, cardiac mortality was found to be increased when followed up 6 years later, regardless of the therapy applied.

## Introduction

Coronary artery bypass grafting and percutaneous coronary interventions are effective methods of myocardial revascularization for patients with coronary artery disease. The population of patients eligible for revascularization has changed in recent years, in regard to significantly increased patient age, number of associated diseases, and number of patients with diabetes. These factors have been reported to exacerbate the development of atherosclerosis [1]. As a result, patients qualified for standard revascularization procedures are more likely to have severe atherosclerotic lesions present in their coronary arteries [2].

Coronary artery disease involving heavily calcified lesions has been associated with worse short- and long-term outcomes after percutaneous coronary intervention [3, 4]. The presence of moderate to severe target lesion calcification has been identified by multivariable risk analysis as an independent predictor of 1-year definite stent thrombosis (hazard ratio [HR] 1.62) and ischemic target lesion revascularization (HR 1.44) [5]. Similar outcomes have been observed in coronary bypass grafting operations, as patients with calcified coronaries had increased rates of mortality and death from myocardial infarction (MI) at 5-year follow-up when compared to patients without calcified coronary arteries [6]. Severe lesion calcification has also been identified as an independent predictor of increased all-cause mortality (HR 1.39) by multivariate Cox regression analysis [6].

Severely calcified lesions make it difficult to complete the distal anastomosis of the graft to the target coronary artery. As a result, there has been an increase in number of patients in whom it is not possible to perform a complete revascularization. Relative to incomplete revascularization, complete revascularization has been associated with lower long-term mortality (risk ratio [RR] 0.71), decreased rates of myocardial infarction (RR 0.78), and repeat coronary revascularization (RR 0.74) [7]. For this reason, clinicians are in search of new methods for the treatment of coronary artery disease in order to achieve complete revascularization and improve patient outcomes.

Transmyocardial laser revascularization (TMLR) is an approved surgical procedure that involves the creation of transmural laser channels within regions of ischemic myocardium. TMLR is performed with a low powered Holmium:YAG laser that delivers precise laser energy directly to the target area of the heart muscle. During TMLR, the surgeon uses one of the CardioGenesis flexible, fiber optic handpieces to deliver precise bursts of Holmium:YAG laser energy directly to the area of the heart muscle that is suffering from ischemic heart disease. It takes approximately 6–10 pulses to transverse the myocardium and creates channels 1 mm in diameter. More than 50,000 TMLR procedures have been performed in more than 38 countries. Over the past two decades, results from prospective and retrospective studies on TMLR have provided data supporting the safety and effectiveness of TMLR when performed as sole therapy, as well as combination therapy with CABG in patients not amenable to traditional methods of surgical or percutaneous revascularization [8–10].

In consideration of the reported favorable outcomes of short-term CABG plus TMLR therapy, it was hypothesized that TMLR in combination with CABG can improve late survival when compared to TMLR or CABG alone group.

The aim of this study was to evaluate the actuarial 10-year survival rate when patients with severe disseminated coronary atherosclerosis were treated with CABG and TMLR.

## Materials and methods

Between 1997 and 2002, 86 patients with three-vessel ischemic heart disease and (diffuse) CAD with severe (coronary) lesion calcification underwent TMLR and/or coronary artery bypass grafting at our institution. Coronary calcifications were stratified visually into severe, moderate, and none to mild using readily apparent densities noted within the vascular wall at the stenosis level. Moderate lesion calcification was defined as radiopaque densities noted during the cardiac cycle involving only one side of the vascular wall, whereas severe lesion calcification was defined as radiopaque densities noted without cardiac motion before contrast injection generally involving both sides of the arterial wall [11].

Diffuse CAD was defined as follows: length of significant stenosis ≥ 20 mm, multiple significant stenosis (≥ 70% narrowing) in the same artery separated by segments of apparently normal (but probably diseased) vessel, and significant narrowing involving the whole length of the coronary artery [12].

All patients (*n* = 86) were scheduled to undergo CABG based upon a previous diagnosis of disseminated coronary atherosclerosis and presence of severe angina symptoms. CABG was performed when possible to create anastomosis (i.e., calcification did not prohibit creation of complete anastomosis). The gold standard in coronary artery bypass grafting was performed by grafting the left internal thoracic artery to the left anterior descending arteries and the remaining anastomosis using venous material. TMLR was performed using a Holmium:YAG laser with an average of 13.8 channels per heart wall. TMLR was performed with a low powered Holmium:YAG laser that delivers precise laser energy directly to the target area(s) of the heart muscle. During TMLR, the surgeon used a CardioGenesis fiber optic handpiece to deliver precise bursts of Holmium:YAG laser energy directly to an area of heart muscle that was suffering from ischemic heart disease. Approximately 6–10 pulses were required to transverse the myocardium and create channels one-millimeter in diameter. Patients received only TMLR in the event where CABG could not be performed due to technical difficulties associated with the graft.

All patients underwent TMLR, but not all patients underwent CABG. A total of 77 patients underwent both TMLR + CABG. Nine patients underwent only TMLR therapy due to technical issues associated with the CABG procedure encountered intra-operatively. Baseline patient demographics and operative procedure information are presented in Table 1. No serious adverse event was associated with the use of the medical laser in the perioperative period.

**Table 1:** Baseline patient demographics and procedure details

Variable	*n* (%), All patients (*n* = 86)
Male, *n* (%)	72 (83.7%)
Mean pre-op ejection fraction^a^ (range)	51.9% (36–70)
Medical history	
HA (arterial hypertension)	42/69 (60.9%)
COPD (chronic obstructive pulmonary disease)	1/69 (1.5%)
DM (diabetes)	13/66 (19.7%)
Chronic kidney disease	2/67 (2.9%)
ST post-MI	50/70 (72%)
Procedure details	*n* (%)
TMR + CABG	77/86 (89.5%)
*CABG Characteristics*	
Total number of CABG grafts	121
Mean number of grafts per patient (range)	1.5 (1–3)
1 graft, *n* (%)	36/77 (46.8%)
2 grafts, *n* (%)	38/77 (49.4%)
3 grafts, *n* (%)	3/77 (3.9%)
Grafted Vessels	
Left main coronary artery (LM), *n* (%)	2 (2.5%)
Left anterior descending artery (LAD), *n* (%)	44 (57.1%)
Marginal branch from circumflex artery (Mg), *n* (%)	34 (44.2%)
Diagonal branch from LAD (Dg), *n* (%)	13 (16.9%)
Right posterior descending from right coronary artery (RPD), *n* (%)	16 (20.8%)
Right coronary artery (RCA), *n* (%)	12 (15.6%)
TMR Only	9/86 (10.5%)
TMLR laser characteristics	Mean (range)
Total TMLR channels per patient	23.4 (5–43)
TMLR channels per heart wall^b^	7.8 (13.8)
Anterior wall^c^	12.5 (5–20)
Lateral wall^d^	13.7 (6–28)
Posterior wall^e^	14.8 (5–28)
Intervention(s) performed according to heart wall	*n* (%)
All Heart Walls	86 (100%)
Combined group (combined TMLR + CABG on any heart wall)	46/86 (53.5%)
TMLR or CABG only (no combined TMRL + CABG on any heart wall)	40/86 (46.5%)
Anterior wall intervention	80/86 (93.0%)
Combined therapy group (TMR + CABG), *n* (%)	8/80 (10%)
Single therapy group (TMR or CABG only), *n* (%)	72/80 (90%)
Lateral wall intervention	64/86 (74.4%)
Combined therapy group (TMR + CABG), *n* (%)	19/64 (29.7%)
Single therapy group (TMR or CABG only), *n* (%)	45/64 (70.3%)
Posterior wall intervention	69/86 (80.2%)
Combined therapy group (TMR + CABG), *n* (%)	19/69 (27.5%)
Single therapy group (TMR or CABG only), *n* (%)	50/69 (72.5%)

^a^Patients (54/86 (62%)) with pre-operative EF reported

^b^Number of channels not reported for all patients

^c^Number of channels reported for 35/44 patients who received channels on anterior wall

^d^Number of channels reported for 36/49 patients who received channels on lateral wall

^e^Number of channels reported for 55/60 patients who received channels on posterior wall

In this series, 46 patients underwent combined CABG plus TMLR on at least one heart wall (“combined therapy group”), while 40 did not receive combined treatment on any heart wall (“single therapy group”). Patients may have undergone concomitant TMLR + CABG with the CABG and TMLR procedures performed on separate heart walls. Results were further analyzed according to procedure type (CABG only, TMLR only, CABG plus TMLR, none) performed on each of the three heart walls (anterior, lateral, or posterior). There were 80 out of the 86 total patients who underwent procedures on the anterior heart wall (*n* = 8 combined therapy group, *n* = 72 single therapy group), while 64 patients had interventions on the lateral wall (*n* = 19 combined therapy group, 45 single therapy group), and 69 patients underwent interventions on the posterior heart wall (*n* = 19 combined therapy group, *n* = 50 single therapy group).

Since descriptive analysis revealed differences in 10-year survival and cardiac mortality only for the evaluation of the intervention performed on the anterior heart wall, this approach was further investigated and patients were divided into two subgroups. The first subgroup composed of patients who underwent combined therapy (CABG and TMLR) on the anterior heart wall, while the second involved the same parameters, but with single therapy (CABG or TMLR only).

Baseline patient demographics for anterior wall intervention are presented in Table 2. No statistically significant difference was observed between the groups who received combined and single therapy in patients with intervention at the anterior heart wall.

**Table 2:** Anterior wall intervention—baseline patient demographics

Variable	*n* (%), All patients (*n* = 86)	*n* (%), Anterior combined therapy group (*n* = 8)	*n* (%), Anterior single therapy group (*n* = 72)	*p*
Male, *n* (%)	72 (83.7%)	6 (75%)	60 (83.3%)	0.55
Age	59 ± 6	55 ± 4	60 ± 9	0.11
Medical history				
HA (arterial hypertension)	42/69 (60.9%)	6/8 (75%)	36/60 (60%)	0.55
COPD (chronic obstructive pulmonary disease)	1/69 (1.5%)	0/8 (0%)	1/60 (1.7%)	0.79
DM (diabetes)	13/66 (19.7%)	0/8 (0%)	11/58 (19%)	0.33
Chronic kidney disease	2/67 (2.9%)	0/8 (0%)	2/58 (3.5%)	0.7
ST post-MI	50/70 (72%)	6/8 (75%)	54/60 (90%)	0.35
Mean pre-op ejection fraction (range)	51.9% (36–70)	55.5% (45–60)	51.9% (36–70)	0.53

The study is a retrospective evaluation of a long-term follow-up of patients who underwent the appropriate procedure which was agreed at the time of intervention. All patients were operated in our center and remained under our supervision and voluntarily showed for follow-up appointments at our outpatient clinic. All patients were consented by writing to include their case into the analysis. The study was approved by the Bioethics Committee (L.dz.OIL/KBL/OIL/11/216).

### Statistical analysis

Statistical analyses were performed using STATISTICA 10. Categorical data were compared by the chi-square test or Fisher exact test if the expected number of observations in any cell was < 5. Continuous data are presented as mean ± SD or median interquartile range and were compared using the analysis of variance or Kruskal-Wallis test. The long-term survival was estimated using Kaplan-Meier time-to-event methodology and was compared using the F Cox test. Multivariable regression analysis using Cox proportional hazard models to assess for independent predictors for probability of survival and for the association between probability of survival and type of procedure was performed during surgery for the whole group and for the anterior heart wall group. Values of *p* < 0.05 were considered to be statistically significant and all *p* values are two-sided.

## Results

The overall mortality rate for all patients was 31%. Median survival for both groups (averaged) was 137 months. The median survival times of 129 and 138 months were for the single therapy and combined groups, respectively (TMLR solo or CABG = 129 months, TMLR + CABG (combined group) = 138 months).

### Ten-year follow-up

A detailed analysis was performed for all patients based upon whether or not they underwent combined procedures of TMLR + CABG on at least one or more wall(s) of the heart. For any heart wall, the cumulative 10-year survival and freedom from cardiac death valued 72.5 and 77.5% in the single therapy group, compared to 78.3 and 80.4% in the combined therapy group (*p* = 0.535 and *p* = 0.739, respectively) (Table 3).

**Table 3:** Ten-year patient survival and freedom from cardiac death according to intervention(s) performed per heart wall

Heart wall	Survival		*p* value	Freedom from cardiac death		*p* value
	Combined therapy group	Single therapy group		Combined therapy group	Single therapy group	
All heart walls (*n* = 86)	36 (78.3%)	29 (72.5%)	0.535	37 (80.4%)	31 (77.5%)	0.739
Anterior wall (*n* = 80)	8 (100%)	52 (72.2%)	0.027	8 (100%)	55 (76.4%)	0.044

Comparison of survival rates for the lateral and posterior wall combined vs. single therapy groups was not statistical significant with rates of 73.7 vs. 73.3% (*p* = 0.976), and 84.2 vs. 72% (*p* = 0.277), respectively. However, when survival rates were compared for the anterior wall combined vs. single therapy groups, the anterior wall combined group significantly improved survival and freedom from cardiac death with rates of 100 vs. 72.2% and 100 vs. 76.4% (*p* = 0.027 and *p* = 0.044), respectively (Table 3).

### Survival benefit at long-term follow-up

**Fig. 1:**
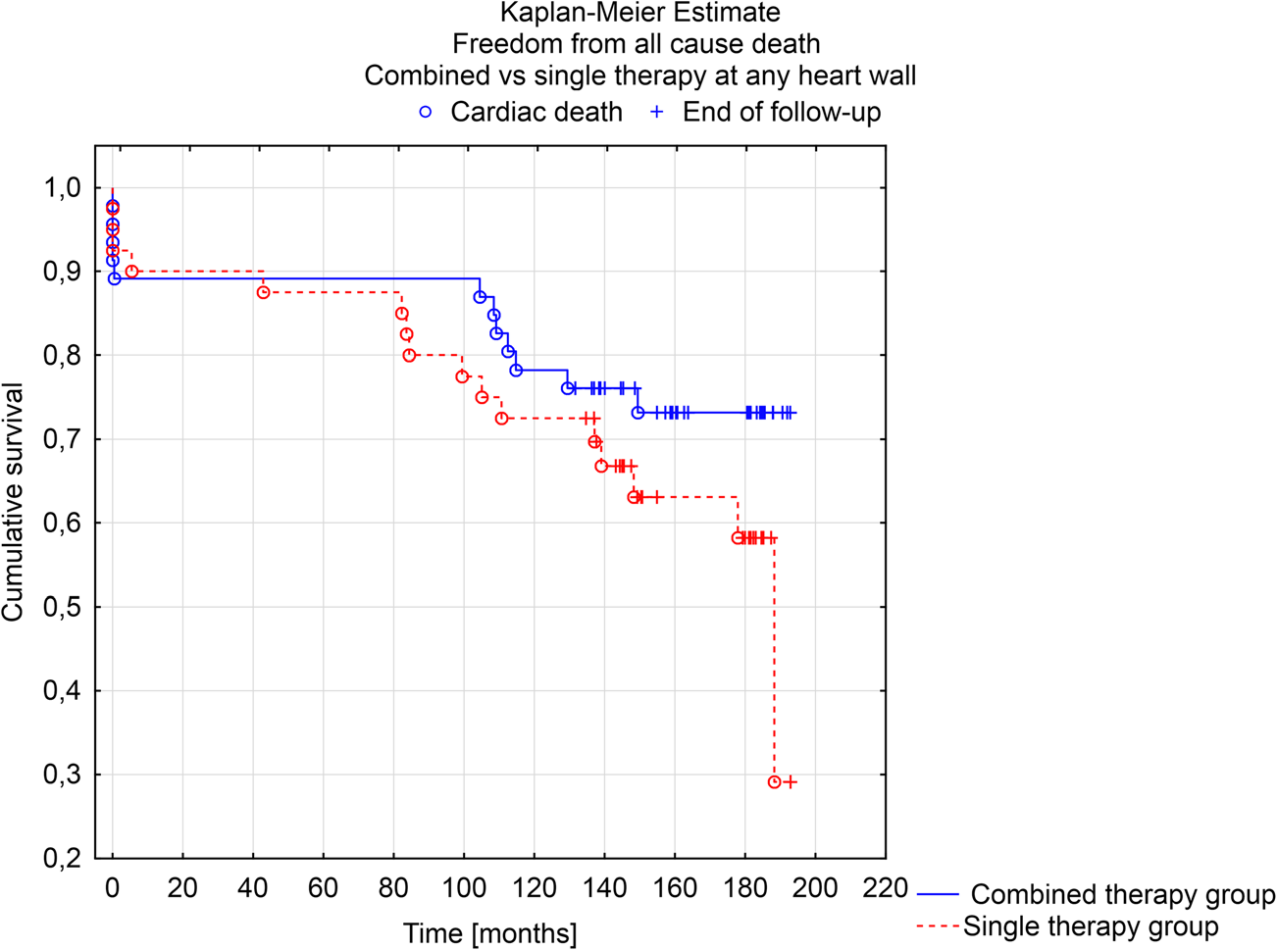
Kaplan-Meier estimate. Freedom from all cause of death in combined vs. single therapy at any heart wall

**Fig. 2:**
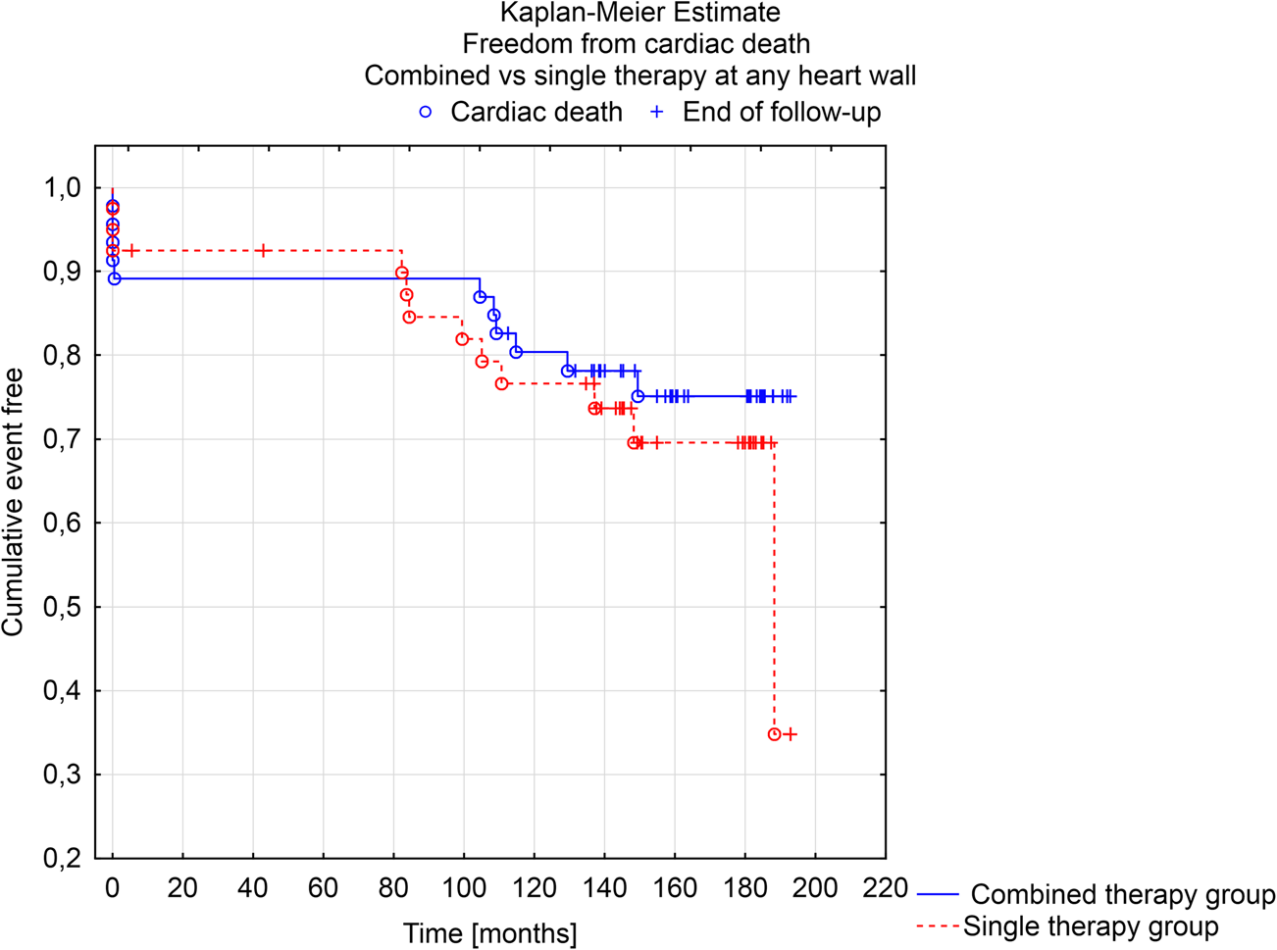
Kaplan-Meier estimate. Freedom from cardiac death in combined vs. single therapy at any heart wall

At Kaplan-Meier analysis, survival at 190 months post-procedure was not significantly different between patients with combined or single therapy group on at least one or more heart wall(s) (F Cox analysis *p* = 0.133). The same results were observed for freedom from cardiac death (F Cox analysis *p* = 0.272) (Figs. 1 and 2).

**Fig. 3:**
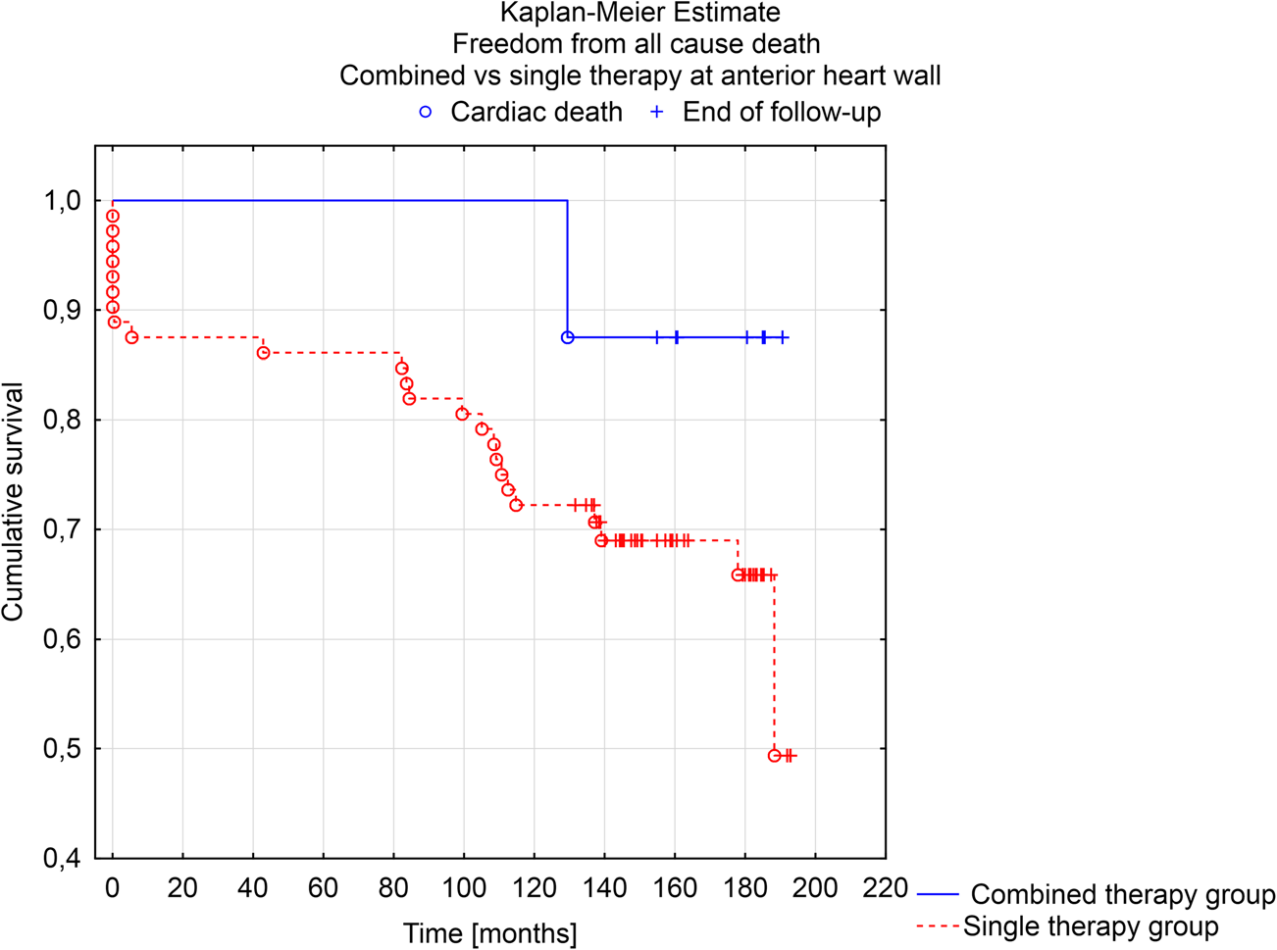
Kaplan-Meier estimate. Freedom from all cause of death in combined vs. single therapy at anterior heart wall

**Fig. 4:**
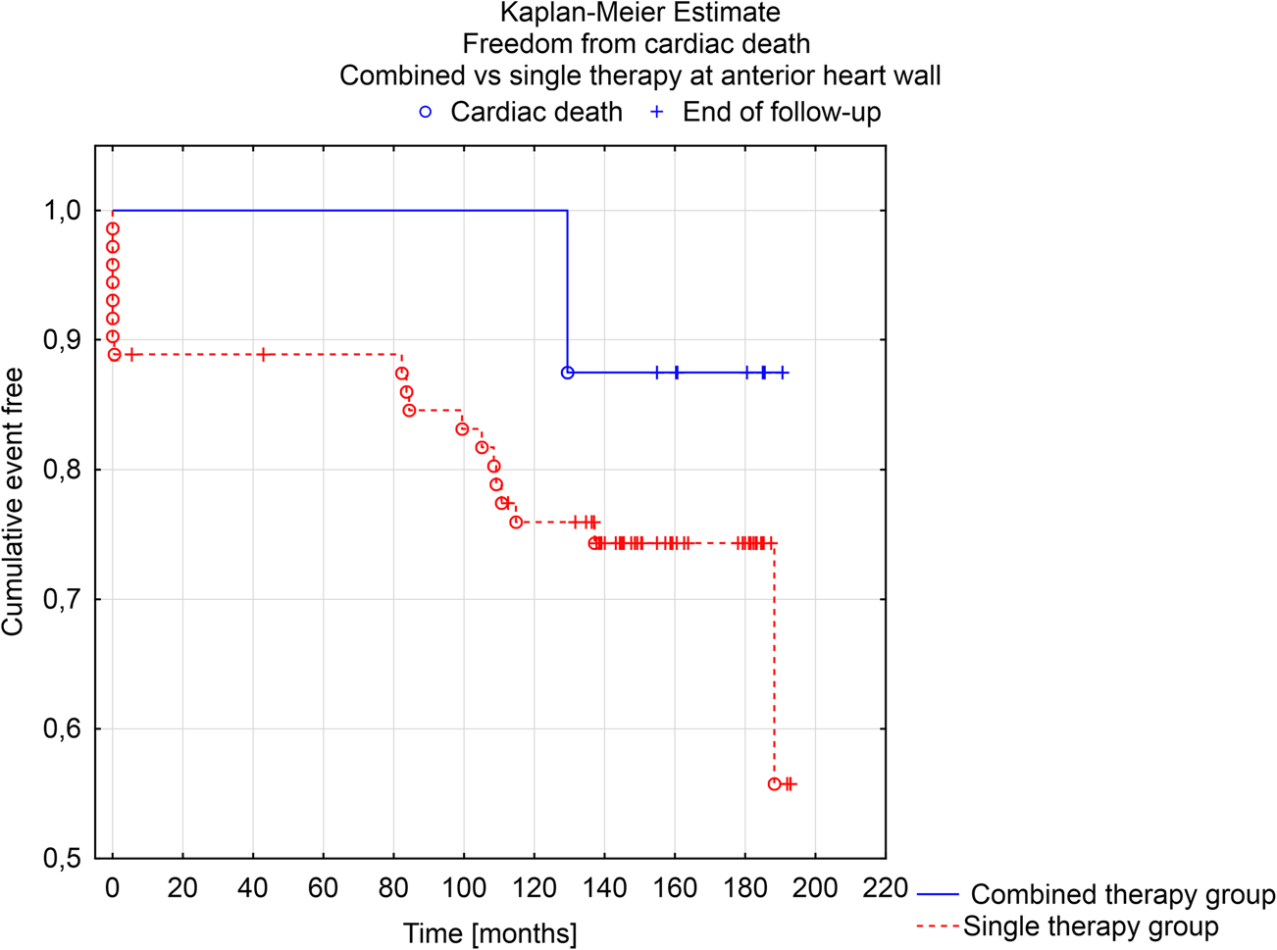
Kaplan-Meier estimate. Freedom from cardiac death in combined vs. single therapy at anterior heart wall

Long-term follow-up results were also further analyzed according to procedure type (combined vs. single therapy group) performed on anterior heart wall. Kaplan-Meier survival analysis showed a trend towards the long-term survival and freedom from cardiac death benefit for combined procedure on anterior heart wall (F Cox analysis *p* = 0.058 and *p* = 0.103) (Figs. 3 and 4).

Moreover, all Kaplan-Meier analysis showed an increased mortality in the early postoperative period. When freedom from all-cause death and cardiac death was observed in all investigated subgroups after 5 to 6 years, survival rates decreased. This deterioration in time was also noted in the subgroup of combined therapy. Thus, a high perioperative mortality was observed following a period of high freedom from cardiac death rate and moderate increase in cardiac mortality after 5 to 6 years of follow-up.

Lack of combined procedures on the anterior wall (odds ratio 3.286, *p* = 0.279) and lack of combined procedures on any heart wall (odds ratio 1.736, *p* = 0.264) were found to be independent predictors of late mortality.

## Discussion

The presence of severe coronary calcification has been associated with “distal” endothelial dysfunction, poor distal bed (quality), and embolic phenomenon associated with coronary calcifications and may lead to increased rate of ischemic outcomes. Severely calcified vessels may result in difficulty performing vascular anastomoses, resulting in prolonged procedural time, suboptimal results, or even failure to achieve a functional graft. In addition, severely calcified coronaries can limit the capacity to achieve complete revascularization, which has been shown to be associated with poor long-term outcome [3, 13, 14].

The Syntax Trial, which reported on the implications of severe coronary calcification in patients undergoing coronary artery bypass surgery trial, found that patients with calcified coronaries, had an increased mortality at 5-year follow-up (17.1 vs. 9.9%, *p* < 0.001). In multivariate Cox regression analysis, severe lesion calcification was also found to be an independent predictor of increased all-cause mortality (hazard ratio 1.39, 95% CI 1.02–1.89; *p* = 0.037) [6]. Severe lesion calcification was associated with an increased mortality in patients undergoing CABG, but it was not an independent predictor of death-MI or MACE. This paradox may be due to CABG allowing perfusion of the healthy coronaries bypassing the diseased arteries, thus minimizing the risk of coronary events due to progressive atherosclerosis.

Only two randomized controlled trials have been performed assessing CABG plus adjunctive TMLR vs. CABG alone [15, 16]. Outcomes from one trial showed reduced operative mortality (1.5 vs. 7.6%, *p* = 0.02) and improved 1-year actuarial survival (95 vs. 89%, *p* = 0.05) for patients that underwent TMLR+CABG, compared to CABG alone [16]. A matched cohort study by Eldaif et al. compared CABG patients with completed revascularization via adjunctive TMLR vs. patients that was incompletely revascularized with CABG alone. Rates of long-term survival were not statistically different; however, TMLR + CABG patients reported sustained symptom improvement with reduction in New York Heart Association classification for class III/IV at 4-year follow-up compared to baseline (*P* < 0.0001) [17]. This study demonstrated TMLR + CABG to be safe and carry no additional risk to patients with no difference in rates of long term-survival when compared to CABG alone. In addition, TMLR was reported to be a beneficial adjunct to CABG to achieve complete revascularization in areas unsuitable for grafts or poor target areas.

The results of this 10 year follow-up showed a vast improvement in survival in patients undergoing surgery at anterior wall with combined TMLR plus CABG, compared to other groups of patients. The anterior wall was an important element responsible for left ventricular systolic function. Left ventricular function impacted overall patient survival, signifying the importance of the intervention performed on the anterior heart wall of patients with severe coronary calcification.

In general, implementation of additional laser revascularization decreased mortality and resulted in a reduction in the severity of myocardial symptoms. The exact mechanism responsible for providing angina relief is still debated. Denervation of the sympathetic nerve fibers innervating the heart is one of proposed mechanism of relief of angina following TMLR. Another proposed mechanism, angiogenesis, or formation of new blood vessels, has shown the strongest experimental and clinical evidence for being the primary basis of angina relief after TMLR. Additionally, upregulation of pro-angiogenic factors, such as vascular endothelial growth factor, fibroblast growth factor 2, and platelet-derived endothelial cell growth factor, was demonstrated in myocardial tissue following TMLR [18–20].

Moreover, in all patients groups, a high perioperative mortality was observed followed by a period of high freedom from cardiac death rate and moderate increase in cardiac mortality after 5 to 6 years of follow-up. Therefore, this study suggests that individuals who undergo incomplete revascularization are in need of close monitoring not only within the early postoperative period, but also in the years post-surgery. This observation presumably arises from the fact that patients who survive perioperative period present with moderate improvement in the left ventricular function, in spite of ongoing ischemic heart failure that exacerbates after few years and results in the late increase of cardiac mortality. Thus, the benefits of applying a combined therapy on any of the heart walls, especially on the anterior heart wall, are observed after 6 years of follow-up. The postoperative improvement in left ventricular function is presumably present after both therapeutic strategies, but in case of a combined therapy applied on any heart wall, the late increase in mortality is less relevant and occurs relatively later post-surgery.

Presented results show that perioperative period is associated with high risk of mortality, both cardiac and non-cardiac. Moreover, as it concludes from the Kaplan-Meier estimate, performing a combined procedure on the anterior heart wall results in lower in-hospital mortality. This observed benefit presumably results from a better protection from the anterior heart wall ischemia when a combined therapy is applied. As the patients who were qualified for this procedure represented an increasing number of individuals with advanced ischemic heart disease and many comorbidities, high perioperative risk was noted prior to the surgery. However, the presented result may improve the strategy to diminish in-hospital mortality in this challenging demography of patients.

More details about myocardial angiogenesis and hemodynamic myocardial function were published by Atluri et al. [21]. In this animal study, author demonstrated an increase in circulating endothelial progenitor cells (EPC) post-TMR. EPCs have the potential to differentiate into endothelial cells and the support network necessary for mature myocardial vasculature. Nuclear factor kB (NFkB) is a potent chemokine and transcription factor that induces angiogenesis. Atluri et al. have shown a dramatic increase in NFkB levels after TMR, whereas this factor was nearly absent in the sham subset. This increase in NFkB levels may play a key role in targeting EPCs to the ischemic myocardium, thereby enhancing EPC mediated angiogenesis and perfusion. Atluri et al. also revealed increases in angiogenesis (18.8 ± 8.7 vessels/high-power field vs. 31.4 ± 10.2 vessels/high-power field, *P* = .02) and perfusion (0.028 ± 0.009 mm^3^ blood/mm^3^ tissue vs. 0.044 ± 0.004 mm^3^ blood/mm^3^ tissue, *P* = .01).

In regard to myocardial contractility, echocardiography showed significant improvement in LV contractility in the ischemic lateral wall post-TMR. Moreover, global wall motion score index showed significant improvements in global myocardial function. These results suggest that myocardial angiogenesis may be important in improvements in patients with anterior wall lesions.

In year 2017, Ahn et al. published meta-analysis of three large studies: SYNTAX, PRECOMBAT, and BEST trials (3280 patients: 1520 patients undergoing CABG and 1692 patients undergoing PCI) [22]. The rate of complete revascularization (CR) was 61.7% (57.2% with PCI and 66.8% with CABG). Overall, 61.7% achieved CR, and patients undergoing CABG more frequently achieved CR than those undergoing PCI. The authors conclude that clinical benefit of CR was less prominent in patients undergoing CABG, as long as the left anterior descending coronary artery was successfully grafted, particularly by using the internal mammary artery [23, 24]. Nevertheless, the meta-analysis showed that CR was associated with a reduction in mortality of 24% compared with IR in patients undergoing CABG. Therefore, CR was considered a goal to reach in both PCI and CABG. Complete revascularization was not always possible due to difficulties such as chronic total occlusion, multiple lesions for PCI, diffuse disease, or a narrowed (2 mm) segment distal to the lesion [25]. In such cases, when complete revascularization cannot be achieved, combined therapy (TMLR + CABG) may be a helpful solution.

### Limitations of the study

This is a single-center, non-randomized study. Data were analyzed retrospectively. No perfusion studies were performed before/after procedures.

## Conclusions

The presence of diffuse atherosclerosis and massive calcification was a proven risk factor for occurrence of MACE in the postoperative period and during follow-up. The combination of TMLR and CABG on the same wall of the heart was beneficial for patients with diffuse atherosclerosis, since the advanced atherosclerotic process prohibited adequate blood supply to the area even when the graft was working properly.

It is hypothesized that TMLR provides additional support to CABG grafts through angiogenesis or the formation of a new network of fine vessels. These neovessels are thought to deliver blood flow to areas of the heart that would not be reached by CABG grafts alone due to the presence of severe atherosclerosis. It is hypothesized that this combination treatment increased the perfusion to grafted areas and was found to be superior to CABG alone, although it was not investigated directly in the presented study.

Combined therapy (TMLR + CABG) provided benefit in perioperative mortality and long-term survival only when provided on the anterior heart wall.

Previous diagnostic methods have been unable to provide clear evidence regarding the mechanism responsible for the effectiveness of TMLR. As a result, TMLR is not a widely accepted treatment for ischemic heart disease. Based on the observations from this novel study, combined TMLR and CABG performed on the anterior heart wall provided good outcomes with improved long-term survival in patients with severe coronary calcification.

For patients with DCA, cardiac mortality increases after 6 years follow-up, regardless of the therapy applied. This increase occurs later in groups of patients who received combined therapy, regardless which heart wall it was.
